# The effectiveness of the behavioural components of cognitive behavioural therapy for insomnia in older adults: A systematic review

**DOI:** 10.1111/jsr.13843

**Published:** 2023-02-19

**Authors:** Declan M. McLaren, Jonathan Evans, Satu Baylan, Sarah Smith, Maria Gardani

**Affiliations:** ^1^ School of Psychology and Neuroscience The University of Glasgow Glasgow Scotland; ^2^ School of Health and Wellbeing The University of Glasgow Glasgow Scotland; ^3^ School of Health in Social Science The University of Edinburgh Edinburgh Scotland

**Keywords:** aged, behavioural therapy, elderly, insomnia, sleep

## Abstract

Insomnia is more prevalent in older adults (> 60 years) than in the general population. Cognitive behavioural therapy for insomnia is the gold‐standard treatment; however, it may prove too cognitively taxing for some. This systematic review aimed to critically examine the literature exploring the effectiveness of explicitly behavioural interventions for insomnia in older adults, with secondary aims of investigating their effect on mood and daytime functioning. Four electronic databases (MEDLINE – Ovid, Embase – Ovid, CINAHL, and PsycINFO) were searched. All experimental, quasi‐experimental and pre‐experimental studies were included, provided they: (a) were published in English; (b) recruited older adults with insomnia; (c) used sleep restriction and/or stimulus control; (d) reported outcomes pre‐and‐post intervention. Database searches returned 1689 articles; 15 studies, summarising the results of 498 older adults, were included – three focused on stimulus control, four on sleep restriction, and eight adopted multicomponent treatments comprised of both interventions. All interventions brought about significant improvements in one or more subjectively measured facets of sleep although, overall, multicomponent therapies demonstrated larger effects (median Hedge's *g* = 0.55). Actigraphic or polysomnographic outcomes demonstrated smaller or no effects. Improvements in measures of depression were seen in multicomponent interventions, but no intervention demonstrated any statistically significant improvement in measures of anxiety. This corroborates with the existing consensus that multicomponent approaches confer the most benefit, and adds to the literature by demonstrating this to be the case in brief, explicitly behavioural interventions. This review guides future study of treatments for insomnia in populations where cognitive behavioural therapy for insomnia is not appropriate.

## 1 INTRODUCTION

### 1.1 Insomnia in older adults

Insomnia disorder is characterised in the Diagnostic and Statistical Manual of Mental Disorders, Fifth Edition (DSM‐V; American Psychiatric Association [APA], 2013) as difficulties initiating or maintaining sleep, or early‐morning awakening. These difficulties must occur despite sufficient opportuning for sleep, three or more times per week, for at least 3 months, and cause distress and/or impairment to daytime functioning. Although estimates of prevalence vary depending on which diagnostic measure is used (6%–48% in the general population; Ohayon, 2002), it is widely accepted that the prevalence of insomnia and insomnia symptoms, specifically issues with sleep maintenance and early‐morning awakening, are greater in older adults than in younger populations (Kocevska et al., 2021; Ohayon, 2002). USA‐based survey reports using DSM‐V criteria estimate the point prevalence of insomnia in older adults to lie between 12% and 20% (Roth et al., 2011), approximately double that seen in the general population (Roth, 2007). Longitudinal observational research in the UK suggests that the prevalence of insomnia increases 1.1 × for every 10 years of lifespan (Morphy et al., 2007), and one study of 6800 adults over 65 years noted an incidence rate of 5% per year (Foley et al., 1999), although these studies are not particularly recent and more up‐to‐date research is needed to determine whether these trends have continued. Ellis et al. (2012) provide some newer insight into this, providing data suggesting the annual incidence of DSM‐5 insomnia to be between 31.2% and 36.6%. However, this study included participants of various ages, and was limited by participant self‐selection.

Insomnia carries an increased risk of many adverse effects in all populations, including older adults. For example, individuals with chronic sleep disturbances experience greater risk of psychological distress and disorders such as depression and/or anxiety, in a bidirectional manner (Buysse et al., 2005). Insomnia in later life has also been associated with disruption in subjective (Wardle‐Pinkston et al., 2019) and objective (Dzierzewski et al., 2018) cognitive abilities. Recent research has also demonstrated that sleep deprivation inhibits the clearance of waste products from human brains (Eide et al., 2021), and meta‐analytic results demonstrate that insomnia is associated with a 1.53 × increased risk of all‐cause dementia (Almondes et al., 2016). Another meta‐analysis also found insomnia to be associated with an 18% increase in risk of developing cardiovascular disease (Zheng et al., 2019), alongside an increased risk of falls and hip fractures (Avidan et al., 2005). Insomnia costs approximately £40bn per annum in the UK alone (Hafner et al., 2016), primarily driven by inpatient care, emergency care and prescriptions (Wickwire et al., 2019). As the proportion of the world's population over 60 years continues to grow, the need for effective and easily disseminated insomnia treatment in older adults is abundantly apparent.

### 1.2 Interventions for insomnia in older adults

In its chronic manifestation, insomnia is unlikely to resolve without intervention (Pigeon, 2010), and evidence suggests chronicity of insomnia may be greater in older adults (McCrae et al., 2003). Treatment can be pharmacological or non‐pharmacological. Physiological changes associated with ageing increase the risk of adverse side‐effects from medications (Patel et al., 2018) and, as such, non‐pharmacological interventions are recommended as the first line of treatment across population groups (National Institutes of Health, 2005) including older adults (Qaseem et al., 2016).

Cognitive behavioural therapy for insomnia (CBT‐I) is the most common of such interventions. CBT‐I is comprised of several distinct components, including Stimulus Control, Sleep Restriction, Sleep Hygiene, Relaxation Training, and Cognitive Therapy (Bootzin & Epstein, 2011; Morin & Espie, 2004). Despite being prescribed less frequently than hypnotic medications, CBT‐I demonstrates efficacy comparable to or exceeding that of pharmacological interventions on insomnia symptoms (Riemann & Perlis, 2009), lasting up to a year post‐treatment (van Der Zweerde et al., 2019), and recent evidence suggests it may also prevent incidence of depression in older adults with insomnia (Irwin et al., 2022). Nonetheless, non‐response rates can be high (Morin et al., 2009), and in some clinical populations such as stroke survivors (Nguyen et al., 2019), the longevity of the effects of CBT‐I may be low. One reason for this could be that the lengthy protocols and cognitive components of CBT‐I may be too taxing for some. Additionally, the intervention also requires adequately trained practitioners, of which there is currently a shortage (Thomas et al., 2016). Previous research has recommended shorter protocols or alternative methods of delivery to ameliorate these issues (Williams et al., 2013). Thus, a comprehensive investigation of easy to disseminate and explicitly behavioural interventions for insomnia is indicated in several populations, and older adults serve as an excellent point of departure.

To do this, it is important to identify which components of CBT‐I are critical, or most effective; of course, this is impossible when CBT‐I is delivered as a multicomponent intervention. To that end, this review examined the efficacy of Sleep Restriction and Stimulus Control, in isolation and in tandem, in treating insomnia and insomnia symptoms in older adults. A secondary aim was to explore the effect of these interventions on mood‐related and daily functioning outcomes. The results of this review should inform clinical practice and guide further research in other populations, and of other behavioural interventions.

## 2 METHODS

The review protocol was registered with International Prospective Register of Systematic Reviews (PROSPERO), registration number CRD42020220663. Preferred Reporting Items for Systematic Reviews and Meta‐analysis (PRISMA; Page et al., 2021) guidance was used in the development of the research question, and in the selection and reporting of studies.

### 2.1 Search procedure and inclusion criteria

A systematic search of MEDLINE—Ovid, Embase—Ovid, CINAHL, and PsycINFO was conducted from inception to 3 December 2020. This was updated on 8 August 2022, to ensure the review was up to date. The search strategy was developed with support from a University of Glasgow Librarian, using keywords and Medical Subject Headings (MeSH), and was adapted for use across all bibliographic databases (the strategy for MEDLINE can be found in Appendix A, Figure A1). The following inclusion criteria were employed.

#### 2.1.1 Population

Common definitions of “Older Adult” were explored. These varied throughout the literature, and some organisations have used a threshold of 60 years or 65 years interchangeably (United Nations, 2019; World Health Organisation, 2018). To include as many relevant studies as possible, the lower threshold of 60 years was adopted. When mixed ages were reported, only those where at least 80% of the sample were ≥ 60 years old were included. To determine this, a normal distribution was assumed, and the *z*‐value for those > 60 years was calculated. Proportions were then derived by selecting the corresponding value from a Standard Normal Table, multiplying by 100, and subtracting from 100.

Studies were included whether insomnia was classified using recognised clinical diagnostic criteria (e.g. DSM, ICSD, ICD) or was self‐reported. Studies where participants presented with sleep and/or circadian disorders distinct from insomnia or were undergoing treatment of severe medical conditions (where the individual was not in remission), for example active cancer treatment, were excluded.

#### 2.1.2 Intervention

Only studies with an explicitly behavioural intervention, including Sleep Restriction and/or Stimulus Control as primary components, were included. Stimulus Control and Sleep Restriction were permitted to be delivered individually or combined in a multicomponent intervention. Studies that included interventions containing different behavioural approaches such as mindfulness were excluded; however, allowances were made for those including sleep hygiene (Posner & Gehrman, 2011), relaxation techniques, and sleep education – provided these were delivered alongside Stimulus Control or Sleep Restriction. Studies where the experimental groups were exposed to any type of cognitive intervention were excluded. That being any intervention where the mechanisms driving change were focused on an individual's thoughts rather than manipulation of the external environment, behaviour change, or reduction of physiological arousal, meaning that employment of CBT‐I was grounds for exclusion.

#### 2.1.3 Comparison/control

Studies with active, passive, or no control groups were included.

#### 2.1.4 Outcomes

The primary outcome of this review was objective and/or subjective changes in sleep and/or insomnia‐related measures, pre‐to‐post intervention. Secondary aims focused on outcomes related to psychological outcomes (depression, anxiety) and daytime functioning. Only studies where primary outcomes reported at pre‐and‐post intervention were included.

#### 2.1.5 Study type

Any type of quantitative, pre‐experimental, quasi‐experimental or experimental study was included. Narrative, critical or systematic reviews, meta‐analyses, commentaries without original results, studies with unpublished data, non‐peer‐reviewed studies, non‐human animal studies and qualitative studies were all excluded. In studies with mixed designs, qualitative results were excluded. No minimum sample size was required.

### 2.2 Study selection and data extraction

Results of database searches were exported to EndNote X9 (version 3.3), and duplicates were deleted. References remaining after this step were uploaded to Rayyan QCRI (Ouzzani et al., 2016) and any remaining duplicates were deleted. Titles and abstracts were screened for inclusion by two independent researchers, optimising selection of appropriate studies (Edwards et al., 2002). The full texts of identified studies were then sourced and screened by the same researchers independently. Disagreements (on average 3%) were resolved by discussion, and by involvement of a third researcher if agreement was not met. Reference sections were also hand searched for studies fitting inclusion criteria.

A study‐specific proforma was created, and two researchers independently extracted data from included studies. These included: citation details, design, control type, sample size, mean age, proportion female, treatment type, number of sessions, delivery method, insomnia definition, and objective and subjective outcome measures at all time points including follow‐up. For outcome measures, numerical values for the mean (*X̅*) and standard deviation (SD) were extracted where appropriate. Where studies reported standard error (SE), this was converted to SD by multiplying by the square root of the sample size. Where these data were not available, authors were contacted to request further information. Where authors provided data with missing pre‐to‐post data for individual participants, these participants were excluded.

### 2.3 Data synthesis and statistical analysis

All statistical analysis was carried out in “R” (R Core Team, 2022), version 4.0.2. Effect sizes for group × time interactions were determined using standardised mean difference (Cohen, 1988), and small sample bias accounted for by calculating Hedges' *g* (Hedges, 1981). These values were computed for each study design using the R package “esc” (Lüdecke, 2019), using the formulae discussed in Wilson (2016). Within‐group effect sizes were only calculated where necessary, to avoid regression to the mean biases. The results are presented as a narrative review, summarising findings, implications and risk of bias.

### 2.4 Risk of bias and quality assessment

Several tools were employed to assess study quality based on its design. Appraisals were carried out independently by two reviewers. Any disagreements were settled by discussion and, if necessary, the opinion of a third party.

For randomised‐controlled trials (RCTs), version 2 of the Cochrane Risk of Bias Tool for Randomized Studies (RoB 2; Higgins et al., 2011) was used. Assessing bias across five domains: the randomisation process, deviations from the intended intervention, missing outcome data, measurement of the outcomes, and selection of the reported result.

Non‐randomised trials were assessed using the Risk of Bias in Non‐randomised Studies of Interventions tool (ROBINS‐I; Sterne et al., 2016). ROBINS‐I assesses risk of bias across seven domains: confounding, selection of participants into the study, classification of interventions, deviations from intended interventions, missing data, measurement of outcomes, and selection of the reported result.

As the ROBINS‐I is not appropriate for appraising single‐armed studies, the Evidence Project Risk of Bias tool was used in these cases (Kennedy et al., 2019). This assesses bias data, random assignment to intervention, random selection of participants, attrition rate, homogeneity of sociodemographic data, and homogeneity of baseline values of outcome measures. A template was created for use of this tool in this study (see Appendix B, Figure A2).

Single‐case experimental design studies were appraised with the Risk of Bias in *N*‐of‐1 Trials (RoBiNT) scale (Tate et al., 2013). This assesses the internal and external validity of a study, and gives a numerical score out of 14 and 16, respectively.

## 3 RESULTS

### 3.1 PRISMA flowchart

**FIGURE 1:**
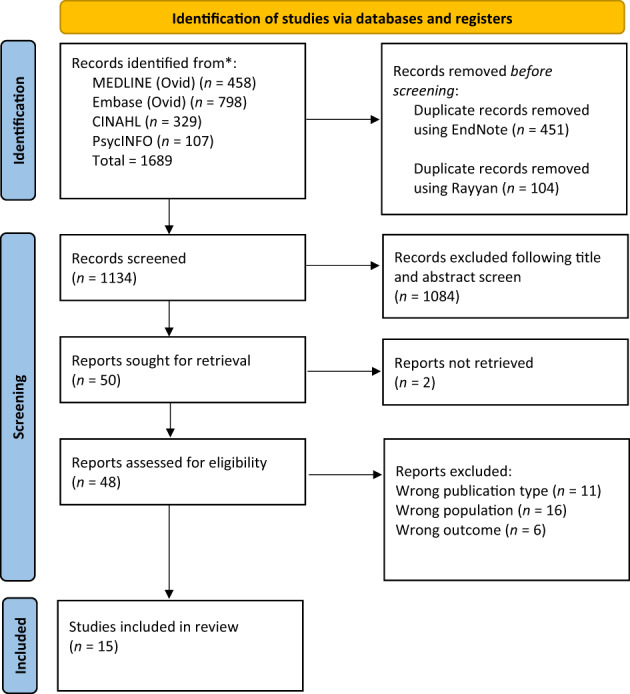
Preferred reporting items for systematic reviews and meta‐analysis (PRISMA) flowchart

Systematic database searches returned 1689 articles. Following title, abstract and full screens, 15 studies were included in the present review. Details of those excluded can be seen in Figure 1. Data requests were submitted for two studies, of which only one responded (Gebara et al., 2019).

### 3.2 Study characteristics

Table 1 provides details of all included studies. All were conducted in the USA, and publication dates spanned 36 years (1983–2019).

**TABLE 1:** Study characteristics

Study	Design	Control	Sample, *N*	Mean age (SD)	% female	Intervention	Sessions	Delivery	Outcome measures	Insomnia definition
Puder et al., 1983	RCT	Delayed treatment	Community based, 16	67.06 (SD Not reported)	81.25	Stimulus control	4	Group	SOL	Author defined SOL > 30 min at least 1 × in a week
Morin & Azrin, 1988	RCT	Imagery training, waitlist	Community based, 27	67.4 (5.6)	62.96	Stimulus control	6	Group	SOL, WASO, TST	Author defined WASO > 30 min per night, at least 3 nights in 1 week, for at least 6 months, with impact to daytime functioning
Friedman et al., 1991	NRT	Relaxation	Community based, 22	70.1 (10.1)	73.30	Sleep restriction	4	Individual	SOL, WASO, TST, SE	Author defined Sleep efficiency < 80% over 2‐week baseline
Bliwise et al., 1995	NRT	Relaxation	Community based including care homes, 32	68.7 (9.7)	68.75	Sleep restriction	5	Individual	SOL, TST	Author defined Self‐reported insomnia
McCurry et al., 1996	SCED	No control	Community based, 4	67.25 (6.08)	75	Multi component	6	Group	PSQI, CES‐D, HDRS, SOL, WASO, TST, SE	Author defined Self‐reported sleep problems ≤3 nights per week
Lichstein et al., 2000	RCT	No treatment	Community based, whilst under care of healthcare provider, 44	68.53 (6.55)	47.73	Stimulus control	4	Individual	IIS, SOL, WASO, SE, TST, SQ, GDS, STAI‐Y	ICSD‐I
Riedel & Lichstein, 2001	Single arm	No control	Community based, 20	67.96 (7.07)	72.73	Sleep compression	6	Individual	SOL, WASO, SE, TST, SQ, ESS	DSM‐IV
Lichstein et al., 2001	RCT	Relaxation, placebo	Community based, 74	68.03 (7.04)	71.62	Sleep compression	6	Individual	IIS, SOL, WASO, SE, TST, SQ, ESS, FSS	ICSD
Germain et al., 2006	RCT	Info only	Community based, 35	70.2 (6.4)	71.43	Multi component	2	Individual	SOL, WASO, SE, TST, PSQI, HARS, HDRS	DSM‐IV
McCrae et al., 2006	SCED	No control	Community based, 4	72.75 (4.86)	100	Multi component	4	Individual	SOL, WASO, TST, SE, SQ, BDI‐ii, GDS, STAI‐Y	ICSD & DSM‐IV
McCrae et al., 2007	RCT	Sleep hygiene	Community based, 20	77.2 (8.0)	65	Multi component	4	Individual	SOL, WASO, TST, SE	ICSD & DSM‐IV
Buysse et al., 2011	RCT	Info only	Community based, 79	71.64 (7.24)	68.40	BBTI	4	Individual	SOL, WASO, TST, SE, SQ, PSQI, HARS, HDRS, ESS	ICSD & DSM‐IV
Tyagi et al., 2014	RCT	Info only	Community based, 79	71.64 (7.24)	68.40	BBTI	4	Individual	SOL, WASO, TST, SE, SQ, PSQI, HDRS	ICSD & DSM‐IV
McCrae et al., 2018	RCT	Self‐monitoring	Community based, 62	69.45 (7.71)	67.74	BBTI	4	Individual	SOL, WASO, TST, SE, SQ, BDI‐ii, GDS, STAI‐Y	DSM‐V
Gebara et al., 2019	RCT	Delayed treatment	Veterans with treatment‐resistant depression, 10	65 (4.01)	27.27	BBTI	4	Individual	ISI, SOL, WASO, SE, PHQ‐9	DSM‐V^1^

Abbreviations: BBTI, Brief Behavioural Treatment for Insomnia; BDI‐ii, Beck Depresion Inventory; CES‐D, Centre for Epidemiologic Studies Depression Scale; DSM‐IV, Diagnostic and Statistical Manual of Mental Disorders, Fourth Edition; DSM‐V, Diagnostic and Statistical Manual of Mental Disorders, Fifth Edition; ESS, epworth sleepiness scale; FSS, Fatigue Severity Scale; GDS, Geriatric Depression Scale; HARS, Hamilton Anxiety Rating Scale; HDRS, Hamilton Depression Rating Scale; ICSD, International Classification of Sleep Disorders; IIS, Insomnia Impact Scale; NRT, Non‐randomised trial; PSQI, Pittsburgh Sleep Quality Index; RCT, randomised‐controlled trials; SCED, single‐case experimental designs; SE, sleep efficiency; SOL, sleep‐onset latency SQ, sleep quality; STAI‐Y, State‐Trait_Anxiety Index; TST, total sleep time; WASO, wake after sleep onset.

#### 3.2.1 Population

The review summarised the data of 498 older adults, all from community‐based samples, recruited via voluntary response sampling. The overall mean age, weighted for sample size, was 69.45 years (range: 65–77.2 years) and average sample size was 35.57. For most studies, insomnia classification was consistent with the most recent DSM or ICSD edition at time of publication. In some cases, author‐defined definitions of insomnia were used (Table 1).

#### 3.2.2 Study design

The entire sample consisted of nine RCTs, one pilot RCT, two non‐RCTs, two single‐case experimental designs (SCEDs) and one single‐armed trial (Table 1).

#### 3.2.3 Intervention

Four studies (Buysse et al., 2011; Gebara et al., 2019; McCrae et al., 2018; Tyagi et al., 2014) investigated Brief Behavioural Treatment for Insomnia (BBTI; Troxel, Germain, & Buysse, 2012; Germain & Buysse, 2011). Tyagi et al. (2014) was a secondary subgroup analysis of Buysse et al. (2011), exploring nocturia's impact on treatment outcome. A further four studies (Germain et al., 2006; McCrae et al., 2006; McCrae et al., 2007; McCurry et al., 1996) were multicomponent studies studying variations of Sleep Restriction, Stimulus Control, Sleep Hygiene, and psychoeducation. Four studies looked at Sleep Restriction, but different methods of restricting time in bed were employed. Friedman et al. (1991) and Bliwise et al. (1995) used conventional Sleep Restriction, where time in bed is abruptly curtailed to match total sleep time (TST; plus a set time to allow for sleep onset) and slowly increased as sleep efficiency increases, whereas Riedel & Lichstein et al. (2001) and Lichstein et al. (2001) employed a protocol akin to Sleep Compression (Lichstein, Thomas, & McCurry, 2011), where time in bed is reduced incrementally. As these variations both target the same psychological mechanisms contributing to insomnia, and are broadly similar, we deemed it appropriate that they be combined for the narrative analysis. The remaining three studies (Lichstein et al., 2000; Morin & Azrin, 1988; Puder et al., 1983) investigated Stimulus Control.

Three studies were delivered in groups (Friedman et al., 1991; McCurry et al., 1996; Puder et al., 1983). The mean number of sessions was 4.53 (SD = 1.12), ranging from 2 to 6, group sizes ranged from 4 to 12. Generally, Stimulus Control and Sleep Restriction studies were published earlier than multicomponent studies.

#### 3.2.4 Comparison

Comparison groups were employed in 80% of included studies, with three studies (McCrae et al., 2006; McCurry et al., 1996; Riedel & Lichstein, 2001) having none. Details of individual comparisons can be seen in Table 1.

#### 3.2.5 Outcomes

Generic details of study‐specific outcomes can be seen in Table 1. Sleep diaries were used in all studies. However, sleep‐onset latency (SOL) was the only outcome to be reported in every trial. Actigraphy was used in three studies (Buysse et al., 2011; McCrae et al., 2018; Tyagi et al., 2014), and polysomnography (PSG) also in three (Buysse et al., 2011; Lichstein et al., 2001; Tyagi et al., 2014). No treatment‐related adverse outcomes were reported in any included studies.

### 3.3 Risk of bias

Risk of bias was assessed according to study design, using the appropriate tool for each study, as detailed in Section 2.4.

#### 3.3.1 Randomised‐controlled trials

**FIGURE 2:**
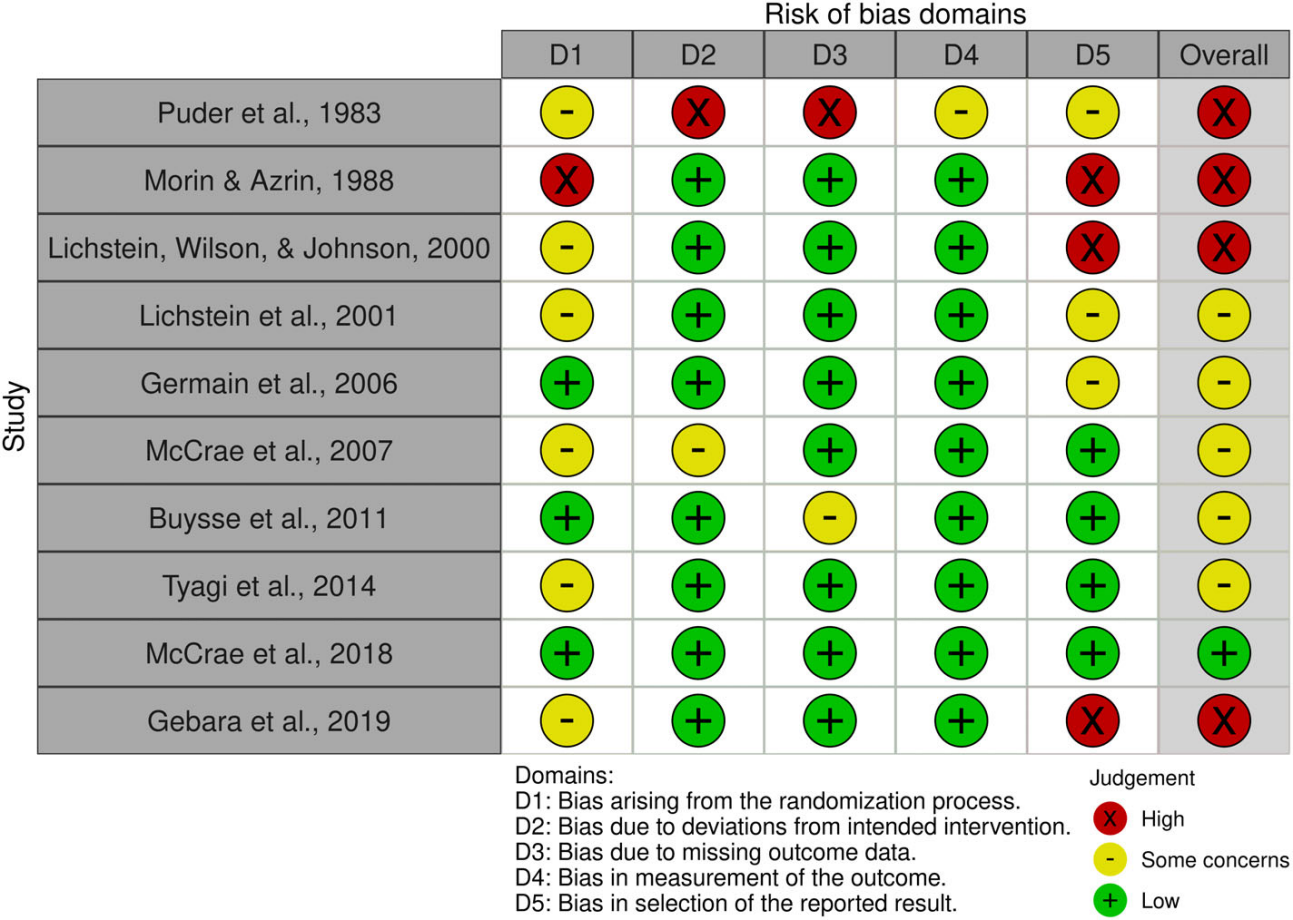
Risk of bias in randomised‐controlled trials (RCTs)

Excluding Gebara et al. (2019), the quality of RCTs generally improved as the publication date became more recent, likely down to changes in accepted standard across time. Only one (McCrae et al., 2018) achieved a low risk of bias overall. Poorer scores were mostly due to participants or researchers not being blinded to group allocation. Often the design made this inevitable; however, measures were mostly self‐report, thus limited researcher bias. A traffic‐light plot of risk of bias in RCTs can be seen in Figure 2.

#### 3.3.2 Non‐randomised controlled trials

**FIGURE 3:**
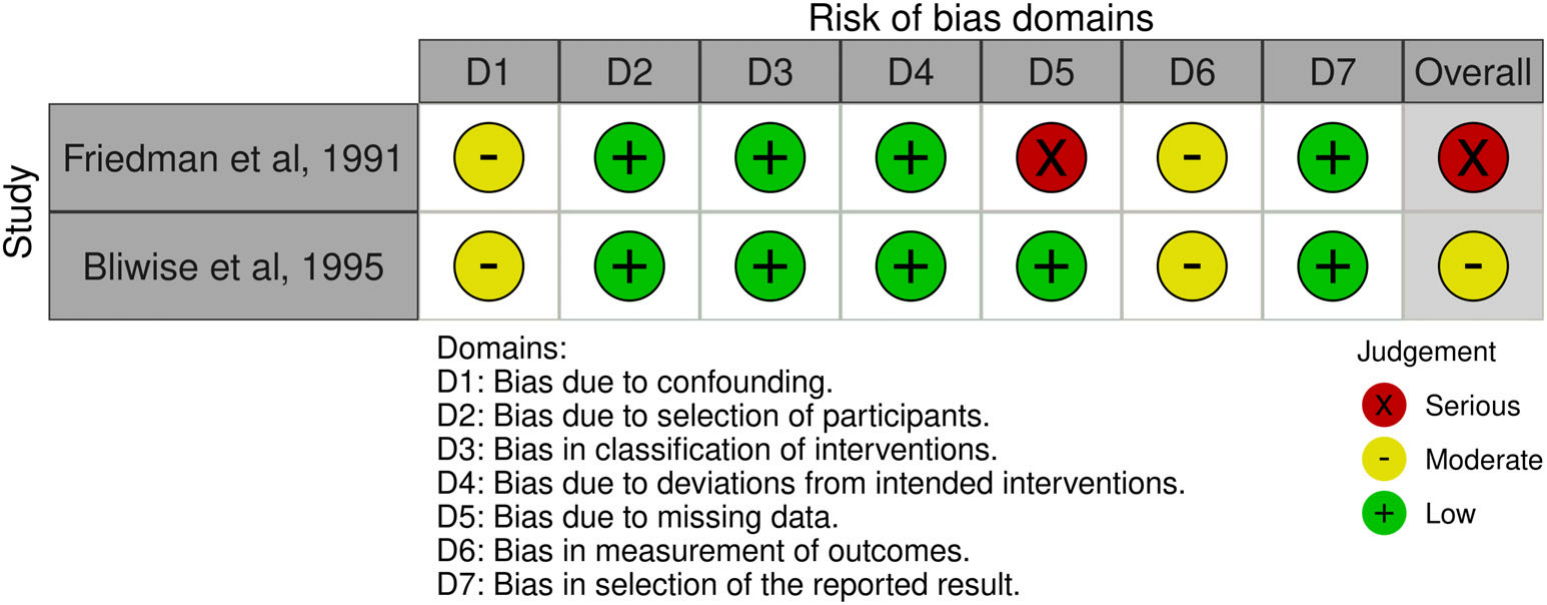
Risk of bias in non‐randomised‐controlled trials (RCTs)

Domain specific risk of bias for non‐RCTs can be seen in Figure 3. Missing data, due to attrition and exclusion of participants on the grounds of missing data, resulted in a serious risk of bias in Friedman et al. (1991). All other domains for non‐RCTs returned a low–moderate risk of bias.

#### 3.3.3 Single‐armed trials

This design undoubtedly introduces the greatest risk of bias overall. Nonetheless, in the interest of rigour, a simple appraisal categorising the presence or absence of specific criteria (Kennedy et al., 2019) was conducted. Many domains were not applicable due to study design, specifically those relating to comparisons of or assignment to groups. Riedel & Lichstein et al. (2001) was a cohort analysis, with pre/post intervention data, and a follow‐up rate > 80%, suggesting a low risk of bias. However, it had no comparison group, and did not randomly select participants, suggesting a high risk of bias. An overview can be seen in Table 2.

**TABLE 2:** Risk of bias in single‐armed studies

	Domain	Response
Riedel & Lichstein, 2001		
	Cohort	Yes
	Control or comparison group	No
	Pre/post intervention data	Yes
	Random assignment of participants to the intervention	NA
	Random selection of participants for assessment	No
	Follow‐up rate of 80% or more	Yes
	Comparison groups equivalent on sociodemographics	NA
	Comparison groups equivalent at baseline on outcome measures	NA

*Note*: a) A response of “yes” suggests a low risk of bias. b) A response of “NA” means this domain is not applicable to this study. c) A response of “No” suggests a high risk of bias.

#### 3.3.4 SCED trials

A high risk of bias was identified in both SCED studies, particularly pertaining to internal validity – specifically, the lack of statistical analysis, and instead presenting only summary data, and no randomisation in sequence of onset of phases between participants. Additionally, although study phases were adequate in length to provide detailed data with five or more data points, only aggregate data were reported (for a summary, see Table 3).

**TABLE 3:** Risk of bias in included SCED studies

	Internal validity							External validity										
Study	Domain 1	Domain 2	Domain 3	Domain 4	Domain 5	Domain 6	Domain 7	Domain 8	Domain 9	Domain 10	Domain 11	Domain 12	Domain 13	Domain 14	Domain 15	Internal validity	External validity	Total
McCurry et al., 1996	1	0	0	0	1	0	0	1	0	1	2	1	0	0	0	2	5	7
McCrae et al., 2006	1	0	0	0	1	0	0	2	1	2	2	1	0	0	1	2	9	11

*Note*: A lower value represents a greater risk of bias. Domain 1: Design with Control; Domain 2: Randomisation; Domain 3: Sampling of Behaviour; Domain 4: Blinding of People Involved in Intervention; Domain 5: Blinding of Assessor(s); Domain 6: Interrater Agreement; Domain 7: Treatment Adherence; Domain 8: Baseline Characteristics; Domain 9: Setting; Domain 10: Dependent Variable (Target Behaviour**);** Domain 11: Independent Variable (therapy/intervention); Domain 12: Raw Data Record; Domain 13: Data Analysis; Domain 14: Replication; Domain 15: Generalisation.

### 3.4 Synthesis of results

This review primarily aimed to explore the effectiveness of behavioural interventions to improve insomnia in older adults, and secondarily to investigate their impact on mood and daily functioning.

Data were synthesised with studies grouped by intervention. Estimates of effect size and confidence intervals are presented in Tables 4, 5, 6, 7, excluding for SCED studies – their design does not allow for the simple computation of effect sizes, and they are discussed separately. Initially it was intended that data of similar studies be pooled for meta‐analysis; however, the heterogeneity of designs and outcomes prevented this. Studies varied across design (thus also effect size calculations), which outcomes were measured and how, whether participants were permitted to continue taking hypnotic medication, whether participants could have insomnia comorbid with another psychiatric or somatic condition, delivery methods, number of sessions, frequency of sessions, how insomnia was defined or whether difficulties were specifically with sleep onset or maintenance, and comparison groups. In some instances, such as in stimulus control, only studies with a high risk of bias could be pooled and, as this could lead to misleading results, it was deemed inappropriate by the researchers. Two studies appeared similar enough to be meta‐analysed, but a meta‐analysis of so few studies would only be appropriate if using a fixed‐effects model. Due to variability between the studies, and the fact that we would intend that the findings be generalised beyond just these two studies, a fixed‐effects model would not be appropriate to generate meaningful inferences in this case.

**TABLE 4:** Effect size estimates for stimulus control studies

Study	Experimental *n*	Comparison *n*	Outcome measure	Hedge's *g*	95% CI	Follow‐up Hedge's *g*	Follow‐up 95% CI	Design
Puder et al., 1983	9	7	SOL	0.92	[−1.16, 3.00]			RCT
Morin & Azrin, 1988 (Treatment versus Imagery Training)	9	8	SOL	0.15	[−1.76, 2.07]	0.49	[−1.56, 2.53]	RCT
			WASO	0.49	[−1.43, 2.41]	0.83	[−1.11, 2.77]	
			TST	−0.77	[−2.71, 1.17]	−0.80	[−2.74, 1.15]	
Morin & Azrin, 1988 (Treatment versus Control)	9	10	SOL	−0.16	[−2.01, 1.69]			RCT
			WASO	0.98	[−0.87, 2.83]			
			TST	−0.96	[−2.80, 0.87]			
Lichstein et al., 2000	23	21	IIS	0.23	[−0.96, 1.41]	0.36	[−0.83, 1.54]	RCT
			SOL	0.12	[−1.11, 1.35]	0.45	[−0.78, 1.68]	
			WASO	0.45	[−0.74, 1.64]	0.43	[−0.77, 1.62]	
			SE	−0.48	[−1.67, 0.71]	−0.58	[−1.78, 0.63]	
			TST	−0.14	[−1.33, 1.04]	−0.30	[−1.49, 0.89]	
			SQ	−0.60	[−1.79, 0.59]			
			GDS	0.12	[−1.06, 1.31]	0.00	[−1.19, 1.19]	
			STAI‐Y	0.43	[−0.77, 1.63]	0.36	[−0.83, 1.55]	

*Note*: Negative value denotes an increase over time.

Abbreviations: CI, confidence interval; GDS, geriatric depression scale; IIS, insomnia severity index; RCT, randomised‐controlled trial; SE, sleep efficiency; SOL, sleep‐onset latency; SQ, sleep quality; STAI‐Y, state‐trait‐anxiety inventory; TST, total sleep time; WASO, wake after sleep onset.

**TABLE 5:** Effect size estimates for sleep restriction studies

Study	Experimental *n*	Comparison *n*	Outcome measure	Hedge's *g*	95% CI	Follow‐up Hedge's *g*	Follow‐up 95% CI	Design
Friedman et al., 1991	10	12	SOL	0.28	[−1.46, 2.02]	0.27	[−1.57, 2.10]	NRT
			WASO	0.50	[−1.27, 2.27]	0.21	[−1.49, 1.92]	
			SE	−1.26	[−3.02, 0.51]	−0.98	[−2.72, 0.77]	
			TST	0.12	[−1.57, 1.82]	−0.41	[−2.10, 1.28]	
Bliwise et al., 1995	16	16	SOL	0.22	[−1.25, 1.70]	0.19	[−1.37, 1.75]	NRT
			TST	−0.18	[−1.59, 1.24]	−0.56	[−1.96, 0.84]	
Riedel & Lichstein, 2001 * +	20		SOL	0.51	[−0.11, 1.13]			Single‐arm
			WASO	0.88	[0.25, 1.52]			
			SE	−0.71	[−1.34, −0.09]			
			TST	0.08	[−0.52, 0.69]			
			SQ	−0.97	[−1.61, −0.33]			
			ESS	0.07	[−0.53, 0.68]			
Lichstein et al., 2001 (Treatment versus control)+	24	23	IIS	−0.09	[−1.23, 1.06]	−0.41	[−1.56, 0.75]	RCT
			SOL	0.04	[−1.14, 1.22]	0.49	[−0.69, 1.66]	
			WASO	0.06	[−1.09, 1.21]	0.45	[−0.70, 1.60]	
			SE	0.17	[−0.99, 1.33]	−0.29	[−1.43, 0.86]	
			TST	0.85	[−0.32, 2.03]	0.07	[−1.09, 1.22]	
			SQ	−0.41	[−1.56, 0.74]	−0.78	[−1.94, 0.38]	
			PSG SOL			−0.17	[−1.35, 1.01]	
			PSG WASO			0.23	[−0.92, 1.38]	
			PSG TST			−0.52	[−1.67, 0.64]	
			PSG SE			−0.47	[−1.62, 0.69]	
			ESS	−0.09	[−1.23, 1.06]	0.05	[−1.09, 1.20]	
			FSS	0.25	[−0.89, 1.40]	−0.06	[−1.21, 1.08]	
Lichstein et al., 2001 (Treatment versus Relaxation Training)+	24	27	IIS	−0.35	[−1.45, 0.75]	−0.34	[−1.44, 0.77]	RCT
			SOL	0.03	[−1.10, 1.16]	0.24	[−0.89, 1.36]	
			WASO	0.01	[−1.10, 1.12]	0.37	[−0.74, 1.48]	
			SE	0.10	[−1.01, 1.20]	−0.20	[−1.30, 0.90]	
			TST	0.85	[−0.27, 1.97]	0.30	[−0.80, 1.41]	
			SQ	0.07	[−1.03, 1.17]	−0.31	[−1.41, 0.80]	
			PSG SOL			−0.37	[−1.55, 0.81]	
			PSG WASO			0.25	[−0.86, 1.36]	
			PSG TST			−0.33	[−1.44, 0.77]	
			PSG SE			−0.17	[−1.27, 0.94]	
			ESS	−0.02	[−1.12, 1.08]	−0.09	[−1.20, 1.02]	
			FSS	−0.39	[−1.49, 0.71]	−0.20	[−1.30, 0.90]	

*Note*: a) Negative value denote an increase over time. b) Effect sizes for studies marked with “*” were calculated as within‐group. c) Studies marked with “+” used Sleep Compression as opposed to Sleep Restriction.

Abbreviations: CI, confidence interval; FSS, fatigue severity scale; ESS, epworth sleepiness scale; IIS, insomnia impact scale; NRT, non‐randomised trial; PSG, polysomnography; RCT, randomised‐controlled trial; SE, sleep efficiency; SOL, sleep‐onset latency; SQ, sleep quality; TST, total sleep time; WASO, wake after sleep onset.

**TABLE 6:** Effect size estimates for multicomponent studies

Study	Experimental *n*	Comparison *n*	Outcome measure	Hedge's *g*	95% CI	Design
McCrae et al., 2007	11	9	SOL	1.22	[−0.71, 3.14]	RCT
			WASO	0.98	[−0.85, 2.81]	
			TST	0.20	[−1.59, 1.99]	
			SE	−1.46	[−3.32, 0.39]	
Germain et al., 2006	17	18	SOL	0.83	[−0.62, 2.29]	RCT
			WASO	0.64	[−0.71, 1.98]	
			SE	−0.68	[−2.02, 0.66]	
			TST	0.18	[−1.14, 1.51]	
			PSQI	1.23	[−0.14, 2.60]	
			HARS	0.49	[−0.88, 1.85]	
			HDRS	0.86	[−0.50, 2.22]	

*Note*: a) Negative value denotes an increase over time. b) Multicomponent SCED studies are not included in this table.

Abbreviations: CI, confidence interval; HARS, hamilton anxiety rating scale; HDRS, hamilton depression rating scale; PSQI, pittsburgh sleep quality index; RCT, randomised‐controlled trial; SE, sleep efficiency; SOL, sleep‐onset latency; SQ, sleep quality; TST, total sleep time; WASO, wake after sleep onset.

**TABLE 7:** Effect size estimates for BBTI studies

Study	Experimental *n*	Comparison *n*	Outcome measure	Hedge's *g*	95% CI	Follow‐up Hedge's *g*	Follow‐up 95% CI	Design
McCrae et al., 2018	32	30	SOL	0.48	[−0.53, 1.49]	0.41	[−0.60, 1.43]	RCT
			WASO	0.63	[−0.38, 1.63]	0.79	[−0.22, 1.80]	
			TST	0.15	[−0.85, 1.14]	−0.23	[−1.23, 0.77]	
			SE	−0.61	[−1.61, 0.39]	−0.69	[−1.69, 0.32]	
			SQ	−0.49	[−1.50, 0.51]	−0.69	[−1.70, 0.31]	
			Actigraphy SOL	0.40	[−0.61, 1.41]	0.01	[−0.98, 1.01]	
			Actigraphy WASO	0.58	[−0.42, 1.58]	0.43	[−0.57, 1.43]	
			Actigraphy TST	0.25	[−0.75, 1.25]	0.10	[−0.90, 1.10]	
			Actigraphy SE	−0.36	[−1.35, 0.64]	−0.29	[−1.29, 0.71]	
			BDI‐II	−0.08	[−1.08, 0.92]	0.01	[−0.99, 1.00]	
			GDS	−0.13	[−1.18, 0.93]	−0.10	[−1.16, 0.96]	
			STAI‐Y	−0.17	[−1.17, 0.83]	0.01	[−0.99, 1.01]	
Buysse et al., 2011 *	39	40	SOL	0.89	[−0.01, 1.80]			RCT
			WASO	0.62	[−0.27, 1.51]			
			TST	0.10	[−0.79, 0.98]			
			SE	−0.78	[−1.67, 0.11]			
			SQ	−0.68	[−1.57, 0.21]			
			Actigraphy SOL	0.55	[−0.38, 1.47]			
			Actigraphy WASO	0.34	[−0.55, 1.23]			
			Actigraphy TST	0.58	[−0.31, 1.47]			
			Actigraphy SE	−0.51	[−1.40, 0.37]			
			PSG SOL	−0.23	[−1.11, 0.65]			
			PSG WASO	0.35	[−0.53, 1.24]			
			PSG TST	0.13	[−0.75, 1.01]			
			PSG SE	−0.18	[−1.06, 0.70]			
			PSQI	1.00	[0.10, 1.89]			
			HARS	0.40	[−0.49, 1.29]			
			HDRS	0.69	[−0.20, 1.58]			
			ESS	−0.15	[−1.37, 1.07]			
Tyagi et al., 2014 (participants without nocturia)*	25	24	SOL	0.98	[−0.24, 2.20]			RCT
			WASO	0.47	[−0.66, 1.60]			
			TST	−0.04	[−1.16, 1.08]			
			SE	−0.91	[−2.07, 0.25]			
			SQ	−0.82	[−1.96, 0.32]			
			Actigraphy SOL	0.73	[−0.45, 1.90]			
			Actigraphy WASO	0.75	[−0.40, 1.89]			
			Actigraphy TST	0.81	[−0.33, 1.95]			
			Actigraphy SE	−0.65	[−2.49, 1.20]			
			PSG SOL	0.00	[−1.13, 1.13]			
			PSG WASO	−0.20	[−1.32, 0.92]			
			PSG TST	−0.07	[−1.19, 1.06]			
			PSG SE	0.11	[−1.02, 1.23]			
			PSQI	1.14	[0.00, 2.29]			
			HRSD	0.55	[−0.58, 1.68]			
Tyagi et al., 2014 (participants with nocturia)*	14	16	SOL	0.81	[−0.72, 2.34]			RCT
			WASO	0.83	[−0.63, 2.28]			
			TST	0.32	[−1.12, 1.76]			
			SE	−0.73	[−2.19, 0.74]			
			SQ	−0.34	[−2.02, 1.34]			
			Actigraphy SOL	0.57	[−0.97, 2.12]			
			Actigraphy WASO	0.22	[−1.24, 1.68]			
			Actigraphy TST	0.65	[−0.84, 2.14]			
			Actigraphy SE	−0.71	[−2.17, 0.75]			
			PSG SOL	−0.29	[−1.81, 1.23]			
			PSG WASO	1.20	[−0.28, 2.68]			
			PSG TST	0.60	[−0.87, 2.07]			
			PSG SE	−0.43	[−1.87, 1.02]			
			PSQI	0.74	[−0.71, 2.19]			
			HRSD	0.49	[−1.02, 2.00]			
Gebara et al., 2019	5	5	ISI	0.96	[−0.36, 2.28]			RCT
			SOL	0.14	[−1.10, 1.38]			
			WASO	0.19	[−1.06, 1.43]			
			SE	−0.28	[−1.53, 0.97]			
			PHQ‐9 with sleep	0.57	[−0.70, 1.84]			
			PHQ‐9 without sleep	0.49	[−0.77, 1.75]			

*Note*: a) Negative value denotes an increase over time. b) Studies marked with “*” used the same dataset.

Abbreviations: BDI‐ii, Beck Depression Inventory; CI, confidence interval; ESS, epworth sleepiness scale; GDS, Geriatric Depression Scale; HARS, hamilton anxiety rating scale; HDRS, hamilton depression rating scale; ISI, insomnia severity index; PSG, polysomnography; PHQ‐9, patient health questionnaire; PSQI, pittsburgh sleep quality Index; RCT, randomised‐controlled trial; SE, sleep efficiency; SOL, sleep‐onset latency; SQ, sleep quality; TST, total sleep time; WASO, wake after sleep onset; STAI‐Y, State‐Trait‐Anxiety Inventory.

#### 3.4.1 Stimulus control studies

Effect estimates for stimulus control studies can be seen in Table 4. Studies varied in how outcomes were measured. SOL was recorded via sleep diaries in Puder et al. (1983) and Lichstein et al. (2000). In Morin and Azrin (1988), SOL and wake after sleep onset (WASO) were measured objectively using a “Switch‐Activated Clock”. The participant applied pressure to the switch when retiring to bed, as the participant fell asleep the pressure on the switch was released.

Puder et al. (1983) and Morin and Azrin (1988) focused on sleep‐onset insomnia and sleep maintenance insomnia, respectively. Lichstein et al. (2000), focused on ICSD‐I insomnia comorbid with another medical or psychiatric condition.

##### Subjective sleep‐related outcomes

Stimulus control is designed to reduce the anxiety one may feel around the bed/bedroom, and train the individual to associate the act of going to bed with sleep; thus, primarily targeting SOL (Bootzin & Epstein, 2011). All stimulus control studies demonstrated significant effects on SOL, which were maintained at follow‐up. However, between‐group differences and a large effect size (albeit with a wide confidence interval) were only reported in Puder et al. (1983). In the others, comparison groups demonstrated similar changes, and effect sizes were indicative of a small or no effect.

Morin and Azrin (1988) and Lichstein et al. (2000) also reported significant improvements in WASO, both of which improved at follow‐up. In the former, stimulus control outperformed a waitlist control and imagery training. In the latter, stimulus control did not result in significantly different results to the waitlist control. TST also improved at post‐treatment but did not improve at a 12‐month follow‐up, and no significant between‐group differences existed.

##### Mood‐related outcomes

Pre‐to‐post values for measures of anxiety and depression were only reported in Lichstein et al. (2000); however, Morin and Azrin (1988) do state that stimulus control elicited no significant effect on either outcome, measured by the STAI and BDI, respectively. Lichstein et al. (2000) report the same, although effect sizes suggest a moderate treatment effect on anxiety at post‐treatment and follow‐up.

#### 3.4.2 Sleep restriction studies

Effect estimates for sleep restriction studies can be seen in Table 5. Riedel & Lichstein et al. (2001) was single‐armed, and thus effect sizes were calculated as within‐group, and any comparisons should be drawn with this in mind. Studies differed in implementation of sleep restriction. Firstly, Riedel & Lichstein et al. (2001) and Lichstein et al. (2001) used sleep compression rather than sleep restriction, where time in bed was reduced to baseline TST gradually, rather than immediately. Studies also varied in what sleep efficiency percentage should be regarded as the threshold at which time in bed is increased. Sleep compression studies opted for 90%, and sleep restriction studies 85%. Additionally, Friedman et al. (1991) opted not to decrease time in bed if the sleep efficiency threshold was not met between sessions.

##### Subjective sleep‐related outcomes

Sleep restriction studies (Bliwise et al., 1995; Friedman et al., 1991) demonstrated statistically significant reductions in SOL, which decreased further after 3 months. Between‐group differences were not significant, and when compared against relaxation training comparisons, effect sizes were small. No significant effects were seen on TST at post‐treatment. However, significant improvements were seen after 3 months, with sleep restriction outperforming relaxation in both studies.

As Riedel & Lichstein et al. (2001) was single‐armed, direct comparisons of sleep compression studies could not be drawn; nevertheless, this study did report significant improvements in SOL, with a moderate effect size. However, in Lichstein et al. (2001) where treatment was compared with relaxation and placebo groups, significant improvements were seen from baseline to post‐treatment in all groups. Sleep compression did maintain improvements better after 12 months, demonstrating a moderate effect against the placebo, and a small effect against relaxation training. Sleep compression did not improve TST significantly in either study.

##### Objective sleep‐related outcomes

Lichstein et al. (2001) was the only Sleep Restriction/Compression study to report objective results, that being PSG data. This was recorded over 2 nights at baseline and follow‐up. Authors elected to use data only from night 2 of PSG recording, due to results indicating that sleep was significantly worse on night 1. In comparison, sleep diary data were recorded over 14 nights. PSG data showed no significant change for any variable between baseline and a 12‐month follow‐up.

#### 3.4.3 Multicomponent studies

Effect size estimates for multicomponent studies can be found in Table 6, and specifically BBTI studies can be seen in Table 7. McCurry et al. (1996) focused on dementia caregivers, McCrae et al. (2006) on non‐specific caregivers at different stages of care, McCrae et al. (2007) solely on rural older adults, and Gebara et al. (2019) on veterans with treatment‐resistant depression. Upon receipt of the data for Gebara et al., it was established that authors had amalgamated the immediate and delayed treatment groups together in their final analysis, due to missing data for several participants in the delayed treatment group; this was confirmed by the authors. The decision was made to take the same approach in this study to avoid inflated effect sizes, and effect sizes for this paper were calculated as within‐group. Other multicomponent studies consisted of older adults from the general population.

##### Subjective sleep‐related outcomes

Only SOL, WASO and sleep efficiency were reported in all multicomponent studies. Treatment brought about significant improvements, and outperformed comparisons, on SOL and WASO in all multicomponent studies. Effect sizes in RCTs ranged from moderate to large (SOL median Hedge's *g* = 0.86; WASO median Hedge's *g* = 0.64). Within‐group effect sizes in Gebara et al. (2019) were small for both outcomes (Hedge's *g* = 0.14 and 0.19, respectively). Data from 3 months post‐treatment (McCrae et al., 2018) demonstrated that improvements in SOL and WASO were well maintained. SCED studies (McCrae et al., 2006; McCurry et al., 1996) reported similar findings, with all participants demonstrating improvements in SOL and WASO at post‐treatment. Sleep quality was reported in 5/8 multicomponent studies, and significant effects were seen in all. Sleep quality was assessed in various ways. The Pittsburgh Sleep Quality Index (PSQI; Buysse et al., 1989) was used in three studies (Buysse et al., 2011; Germain et al., 2006; Tyagi et al., 2014), Germain et al. (2006) used a 0–6 self‐report scale, McCrae et al. (2018) used a 0–5 self‐report scale, and Tyagi et al. (2014) also reported a 0–100 self‐report scale. Interestingly, results from this were only significant in the group without nocturia.

Results from Tyagi et al. (2014) suggest that the magnitude of the effect of BBTI on SOL was smaller in the group with nocturia, but still statistically significant. Contrastingly, the size of the effect on WASO was larger in those with nocturia than without. It should be noted that WASO was larger at baseline in people with nocturia (67.02 [SD = 34.17] min versus 44.32 [SD = 30.36] min) than those without.

Multicomponent therapy did not result in any significant effects of time, group or group × time on TST in any RCTs. Results were inconsistent in the SCED trials. Three (75%) participants in McCurry et al. (1996) reported increases in TST, but only one participant (25%) in McCrae et al. (2006) did so, whilst two reported decreases.

##### Objective sleep‐related outcomes

The BBTI brought about statistically significant improvements in actigraphic SOL and WASO in Buysse et al. (2011), with moderate effect sizes. However, Tyagi et al. (2014) demonstrated that these improvements were only seen in those without nocturia. McCrae et al. (2018) was the only other multicomponent study to report actigraphic outcomes, and demonstrated no significant changes in any sleep diary domain.

In Tyagi et al. (2014), participants with nocturia who received BBTI experienced a significant decrease in PSG WASO, where those with nocturia and controls experienced an increase. Note that BBTI participants with nocturia had elevated WASO at baseline (116.99 min [SD = 47.77]) compared with those without nocturia (76.15 [SD = 38.11]). No other significant effects were seen in PSG outcomes.

Apart from McCrae et al. (2018), all multicomponent studies that recorded depression, including both SCED studies, demonstrated significant improvements in depression outcomes across varying self‐report and interview‐based measures. In Tyagi et al. (2014), statistically significant improvements in depression were only seen in participants who received BBTI and had nocturia. Measures of anxiety were reported in four multicomponent trials (Buysse et al., 2011; Germain et al., 2006; McCrae et al., 2006; McCrae et al., 2018). No statistically significant change was found, nor were the results in SCED studies suggestive of an effect.

## 4 DISCUSSION

This systematic review is the first to investigate explicitly behavioural interventions, without any cognitive component, for insomnia in older adults. Specifically, this review aimed to examine the literature detailing the efficacy of Sleep Restriction and Stimulus Control therapies, both isolated and combined, at treating insomnia in older adults. The secondary aim was to explore the effect of these treatments on outcomes of mood and daytime functioning when these were reported. Our findings support the efficacy of these interventions, in alleviating the symptoms of insomnia in older adults. Albeit varying in the domains affected, and in the magnitude of effect, all interventions explored in the present review conferred some benefit to the participants.

### 4.1 Sleep‐related outcomes

Given their importance in the nosology of insomnia, SOL and WASO are of particular interest. Improvements in subjective measures of these domains were demonstrated in all interventions. In isolation, Stimulus Control and Sleep Restriction both demonstrated an effect of time, which were maintained at 3‐month follow‐ups, but generally did not achieve significant between‐group differences. Where they did (Puder et al., 1983; Riedel & Lichstein, 2001), studies were older and contained a higher risk of bias. Contrastingly, multicomponent interventions demonstrated significant group × time effects on subjective SOL and WASO (median Hedge's *g* = 0.62), which were maintained 3 months after treatment ended; the one exception being Gebara et al. (2019). This effect on SOL and WASO was demonstrated to be the case even after just two sessions of a multicomponent intervention (Germain et al., 2006; Hedge's *g* = 0.83 and 0.64, respectively). Effect sizes in studies using solely stimulus control or sleep restriction were generally smaller and notably more heterogenous. Inconsistency across all studies in what measures were used meant that TST was the only other sleep‐related outcome for which data were sufficient to draw inferences. In stimulus control studies, TST increased, but group differences did not exist, nor did improvements continue at a 12‐month follow‐up. All studies with a sleep restriction component, isolated or combined with stimulus control, demonstrated small or no effects on TST immediately post‐treatment, likely as a result simply of the nature of the therapy, initially reducing time in bed, and by extension TST. Where follow‐ups are reported, TST does appear to lengthen, and the size of the effect appears to increase over time after treatment has ended.

Where they are available, objective measures tell a slightly different story than subjective ones. In Tyagi et al. (2014), BBTI demonstrated a large statistically significant effect on PSG WASO in participants with nocturia. No other differential effects were witnessed in any other PSG‐recorded outcomes from any study. Actigraphic data were available only from BBTI studies (Buysse et al., 2011; McCrae et al., 2018; Tyagi et al., 2014), and were generally more consistent with sleep diary data. Treatment brought about improvements in actigraphic SOL and WASO in all cases; however, these improvements were not statistically significant in McCrae et al. (2018), despite outperforming the control group and demonstrating a moderate effect size in both outcomes. It is possible that moderate effect sizes may not be sufficient to reach statistical significance in groups of this size (*n* per group = 31). Note that the confidence intervals for these effect sizes are wide, suggesting a low degree of certainty to the true estimate. Results indicated no significant effect of treatment on actigraphic TST at post‐treatment, except in BBTI participants without nocturia. However, like sleep diary data, actigraphic TST appears to begin to increase at 3‐month follow‐ups.

### 4.2 Mood and daytime functioning‐related outcomes

No measures of mood were reported in any isolated sleep restriction/compression study, and no effect was seen on any measure of daytime functioning. Stimulus control studies reported outcomes on depression, anxiety and measures of insomnia impact, but elicited no effect on any. When combined, stimulus control and sleep restriction appear effective at alleviating depression, but not anxiety. Despite a variety of different measures for both outcomes being employed across all studies, a clear pattern can be seen. Seven multicomponent studies provided data on measures of depression. Of these, six demonstrated significant improvements, with moderate effect sizes. The exception was McCrae et al. (2018); however, average BDI‐II (Beck et al., 1996) and the GDS (Yesavage, 1988) scores at baseline were in the normal range for both groups, thus treatment effect may not have been witnessed simply due to the absence of elevated levels of depression in the sample. Four multicomponent studies provided data on anxiety, of which none demonstrated any significant improvements.

### 4.3 Interpretation of results

The efficacy of CBT‐I is well established, and in myriad different populations (Riemann et al., 2022). As a result, it is recommended as the first line of treatment for insomnia by The American College of Physicians (Brasure et al., 2016; Qaseem et al., 2016), The American Academy of Sleep Medicine (Edinger et al., 2021), The European Sleep Society (Riemann et al., 2017), and The British Association for Psychopharmacology (Wilson et al., 2019). As a result, most contemporary research has focused on CBT‐I, which though effective, can be both financially and temporally costly, and may not be best suited in some clinical populations. For example, two recent studies, one pilot RCT (Nguyen et al., 2019) and one case series (Herron et al., 2018) explored the utility of CBT‐I following a stroke. Although both demonstrated improvements in sleep, mental health, quality of life and fatigue, improvements in insomnia and quality of life were not maintained at follow‐up. Deficits in cognitive ability (Sun et al., 2014) and speech and language (Berthier, 2005) are common following a stroke, and thus the cognitive demand of CBT‐I and the six–eight sessions it is ordinarily delivered in may be too taxing. The renewed interest in unpicking the constituent components of CBT‐I (Riemann et al., 2022), particularly without cognitive facets, may prove fruitful in improving treatment options in this domain. This review demonstrates that explicitly behavioural interventions can elicit moderate to large positive effects on outcomes critical to insomnia disorder. By extension, it has the potential to inform future research into developing interventions for insomnia following a stroke, or in similar populations, without the need for cognitive components. Moreover, by isolating the specific therapies that are advantageous in the treatment of insomnia, we can identify and evaluate the key mechanisms mediating clinical change and begin to explore when monotherapies such as stimulus control and sleep restriction could be more efficient alternatives (Maurer et al., 2021).

In 2021, the American Academy of Sleep Medicine endeavoured to determine the “minimal characteristics” of a CBT‐I intervention, in the general population. The main conclusion was that CBT‐I should be comprised of at least: Sleep Restriction Therapy, Stimulus Control, and some cognitive component (Edinger et al., 2021). In addition to this, they noted that Stimulus Control and Sleep Restriction may be used as a single‐component therapy on a conditional basis. They also note that these therapies may require adaptation or extra support in certain populations, such as older adults (Edinger et al., 2021). Hypnotic medications are recommended only for short‐term use, in instances where CBT‐I fails to work or is unavailable (Riemann et al., 2022), and non‐pharmacological intervention is the preferred choice of most patients (Irwin et al., 2006), thus alternative behavioural options may increase treatment adherence. The results of the present review enhance our understanding of these options in older adults, and demonstrate that effective treatment can be delivered without cognitive therapy in as little as two sessions, with moderate to large effects (Germain et al., 2006).

The findings related to TST are consistent with existing CBT‐I literature (Scott et al., 2022). A diagnosis of insomnia is not contingent upon the duration of sleep, and thus TST is rarely a focus of treatment. Nonetheless, when long‐term outcomes are explored, additional gains in SOL and WASO are infrequently reported; however, TST appears to increase somewhat linearly by about 30–40 min, 3–6 months after treatment (Scott et al., 2022). In one study of 80 adults (> 30 years old), after 6 weeks of CBT‐I, only 45.4% reported a TST that exceeded that of baseline. By 24 months post‐treatment, this had increased to 85.9%, without any additional clinical intervention (Scott et al., 2022). These increases only began to occur 3, 6 and 12 months after treatment. Whether or not participants benefit from increased TST remains unclear; however, this stresses the importance of lengthy follow‐ups to shed more light on the true effects of these interventions.

Similarly, discrepancies between subjective and objective measures, known as “paradoxical insomnia” or “sleep state misperception” are known phenomena in other populations (Riemann et al., 2022). PSG commonly results in less pronounced changes than self‐report measures. For example, previous meta‐analytic results reported average differences in PSG‐recorded TST between people with insomnia and good sleepers of approximately 25 min, whereas subjectively recorded average differences are almost 120 min larger (Baglioni et al., 2014). This remains to be a significant clinical and scientific challenge (Riemann et al., 2022). In all cases in this review, PSG data were collected over 2 nights and, in Lichstein et al. (2001), the first night was ignored to account for “first‐night‐effect” (Agnew Jr et al., 1966). Of course, this provides less data points to average across than the 14 nights measured in sleep diaries and may be a potential explanation for the difference.

The contradictory results exhibited in Gebara et al. (2019) are most likely a result of the study being a pilot, designed to assess feasibility, and not powered to detect differences between groups (Brysbaert, 2019). Pilot studies were included in this review to capture as much relevant literature as possible, but the limitations of their design should be considered, as well as the fact that effect sizes in Gebara et al. (2019) were calculated as within‐group.

### 4.4 Clinical implications and applications

These findings substantiate, add to the existing literature, and are of particular importance in guiding future investigations into treating insomnia in populations for whom cognitive therapy may not be appropriate or optimal. The evidence suggests that explicitly behavioural interventions, even brief ones, can be effective at alleviating the symptoms of insomnia, and consequently depression symptoms, in older adults, making a case for the dissemination of these interventions when CBT‐I is unavailable or inappropriate. Rather surprisingly, all 15 included studies were conducted in the USA. This does limit the cross‐cultural application of the findings, and is perhaps partly explained by studies not published in English being excluded. However, considering insomnia prevalence and that the inclusion criteria were so broad, it was an unexpected finding, and does raise questions as to why more investigation of the behavioural components of CBT‐I in older adults has not been conducted in other countries.

### 4.5 Strengths

Our review has several strengths. Firstly, the protocol for the review was pre‐registered, promoting transparency in our procedures, reducing the potential for bias, and avoiding unintended duplication of research within the field. Additionally, the recently revived interest in unpicking the individual components of CBT‐I to determine their individual utility (Riemann et al., 2022), along with the current focus on improving our understanding of insomnia in older adults, as evidenced by the 300+ active studies listed on clinicaltrials.gov, highlights the timely nature of the present review, and its relevance to the current objectives of the field at large. Moreover, our broad inclusion criteria and comprehensive search strategy lessens the likelihood of relevant literature being missed out. Thus, we can be confident we have explored the entirety, or as close to it as possible, of the existing evidence of the efficacy of behavioural interventions in older adults with insomnia.

### 4.6 Limitations

Some inevitable limitations to this review do exist. Firstly, only papers that were published in English were included. Meaning that any relevant papers published in other languages are therefore not addressed in this review. Moreover, although a great deal of planning and experimentation went into developing search terms for bibliographic database searches, it is of course possible that not all the terminology relevant to this research was used. Thus, any papers published by authors who have not used conventional terminology may have been missed. However, reference sections of included studies were searched, and detailed scoping searches were used to investigate the most appropriate terminology to use.

Two additional field‐wide limitations also exist. Firstly, despite a relatively broad inclusion criteria, we were only able to identify 15 suitable studies. These studies differed widely with regards to insomnia classification, study design, delivery method, outcomes reported, comorbidities, nature of comparison and control groups, duration of treatment, number of therapeutic sessions, length of follow‐up, and at times the interventions themselves. This phenomenon has been noted in previous meta‐analyses exploring Sleep Restriction in general populations (Maurer et al., 2021), and CBT‐I (Mitchell et al., 2019; van Straten et al., 2018). The field‐wide lack of uniformity, particularly in selection of outcome measures, limits what inferences can be drawn. Specific domains where this issue is of particular concern are in the measurement of mood‐related outcomes, and in sleep quality. Seven of the 15 studies did not measure sleep quality at all, those that did either used the PSQI (Buysse et al., 1989) or a self‐report rating scale – either 0–5, 0–6 or 0–100. The PSQI was also only adopted in multicomponent studies, making valid between‐intervention comparisons for this outcome impossible. Moreover, variation in the design of studies included meant effect sizes were calculated differently for different studies, impairing the validity of comparisons between studies. The calculation of within‐group effect sizes negates the benefit of having a control group and is sensitive to influence from pre‐test variability (Dankel et al., 2017). Generally, this, along with concerns highlighted in risk of bias appraisals, identifies field‐wide limitations, and the need for more high‐quality RCTs. More specifically, the variation between studies meant that a meta‐analysis was not possible as part of this review. Nonetheless, it did allow for an exploration offering new information on this topic, and the large amount of data extracted from the included studies allowed for the valid comparison of other outcomes.

Secondly, the sample sizes of many included studies were low. In general, the quality of study tended to increase over time, as did sample size, and this is likely a result of improvements in our understanding of study design and statistical power in psychological research across time. Of course, given how few studies appear to have explored these interventions as monotherapies in older adults, expected effect sizes may not be available. In these circumstances, if we assume *d* = 0.4 to be an acceptable rough estimate of the smallest effect size of interest in psychological science (Brysbaert, 2019), a simple comparison of two within‐subjects conditions would require a minimum of 50 participants to achieve a power of 0.80, increasing into the hundreds as the complexity of the study increases (Brysbaert, 2019). Very infrequently do psychological researchers purposefully run a study that includes more than the minimum number of participants. This is seen as waste. However, the history of the field has demonstrated that this has at times resulted in many underpowered studies (Brysbaert, 2019). Kühberger et al. (2014) demonstrated that the mean effect size found in studies with 50 participants is reliably larger than that in studies of 500 participants. Compounded by publication bias, this can then result in standardised effect sizes of published studies being too large when evidence of an effect is repeatedly demonstrated in small samples (Maxwell, 2004).

The vast majority of included studies (13/15) were largely made up of female participants. Sex differences in the prevalence of insomnia have been widely explored. One recent meta‐analysis of 13 observational studies found a significantly higher prevalence of insomnia in females (odds ratio [OR] = 1.58; 95% confidence interval [CI] = 1.35, 1.85). This, accompanied with the primarily female samples included in this review, may limit the generalisability of the results.

### 4.7 Future directions

The present review highlighted several areas where the empirical literature is lacking in the investigation of behavioural interventions for insomnia in older adults. Firstly, all studies employed community‐based samples, and it would be useful to investigate these interventions in other populations, such as those living in nursing homes. Moreover, results from Tyagi et al. (2014) highlight how the outcomes of interventions can vary in different clinical populations. Future work should aim to delve deeper into this, in different samples. Studies outside of the USA as well as studies exploring the possibility of sex differences in treatment response would be particularly prudent. Additionally, this review focuses on the effect of stimulus control and sleep restriction on outcomes related to sleep and mental health; however, the relationship between disrupted sleep and impaired cognitive function (Deak & Stickgold, 2010) is well established. A comprehensive examination of how behavioural interventions for insomnia may impact this relationship is indicated. In the case of multicomponent interventions including both sleep restriction/compression and stimulus control, researchers should consider experimental designs allowing for the incorporation of both “process” and “outcome” research. It is also important to consider measuring cognition at several points throughout treatment, to account for the possibility of sleep restriction impairing cognition in the short term. With the contemporary approach to healthcare of “precision medicine” (Ginsburg & Willard, 2009) comes a curiosity of how this notion can be applied to sleep medicine. Exploring different combinations of behavioural and cognitive methods to treat insomnia would be a prudent point of departure in determining which is optimal in different clinical populations. An extension to this should then be to determine in which groups, and how, these interventions can be implemented digitally. The feasibility and acceptability of internet‐delivered CBT‐I is already established (Seyffert et al., 2016), but the efficacy of its individual components delivered in this way is yet to be established.

## 5 CONCLUSIONS

For some clinical populations, CBT‐I may be cognitively taxing. By dissecting the individual components on CBT‐I, and removing the cognitive aspects, we can evaluate the key behavioural mechanisms that drive clinical change. Thus, it is imperative that we develop a deeper understanding of how explicitly behavioural interventions elicit change in different clinical populations, allowing us to not only identify the optimal interventions for said groups, but also increase the ease with which effective, evidence‐based treatment for insomnia can be disseminated to those who need it. This is the first systematic review to investigate focused behavioural interventions for insomnia in older adults. The results suggest that Stimulus Control and Sleep Restriction are both effective in alleviating insomnia symptoms in older adults, particularly when combined as a multicomponent therapy. Moreover, these interventions are demonstrated to elicit moderate to large effects on SOL and WASO, after only two therapeutic sessions. Impact on mood is less clear, but it does appear that when combined, stimulus control and sleep restriction can also elicit significant improvements on measures of depression, but not anxiety.

## AUTHOR CONTRIBUTIONS

Declan M. McLaren: conceptualisation, methodology, writing – original draft, project administration, formal analysis, investigation, data curation, visualisation; Jonathan Evans: conceptualisation, methodology, writing – review & editing, project administration, supervision, funding acquisition; Satu Baylan: conceptualisation, methodology, writing – review & editing, project administration, supervision, funding acquisition; Sarah Smith: data curation; Maria Gardani: conceptualisation, methodology, writing – review & editing, project administration, supervision, funding acquisition.

## CONFLICT OF INTEREST STATEMENT

The authors report no conflicts of interest.

## Supporting information


**FIGURE A1** Search strategy for Ovid Medline Electronic Database

**FIGURE A2** The Evidence Project Risk of Bias Appraisal Tool Template

## Data Availability

Data sharing is not applicable to this article as no new datasets were generated during the current study.
