# Supplementary figures and images for: The effectiveness of the behavioural components of cognitive behavioural therapy for insomnia in older adults: A systematic review
Source: J Sleep Res. 2023 Feb 19;32(4):e13843. doi: 10.1111/jsr.13843 (PMC10909422; doi:10.1111/jsr.13843)

***Appendix A***


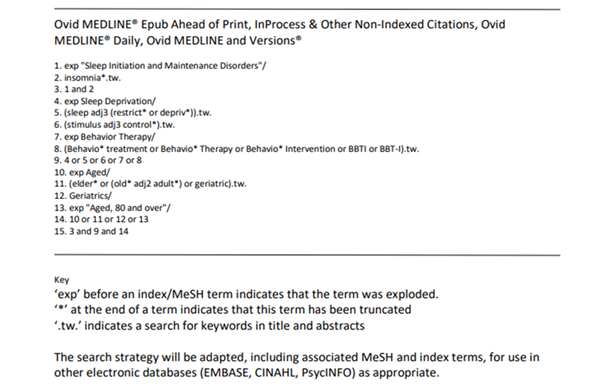


**Figure A1: Search Strategy for Ovid Medline Electronic Database**

Supplement: Supplementary file 1 — FIGURE A1 Search strategy for Ovid Medline Electronic Database [file JSR-32-e13843-s002.docx]

***Appendix B***


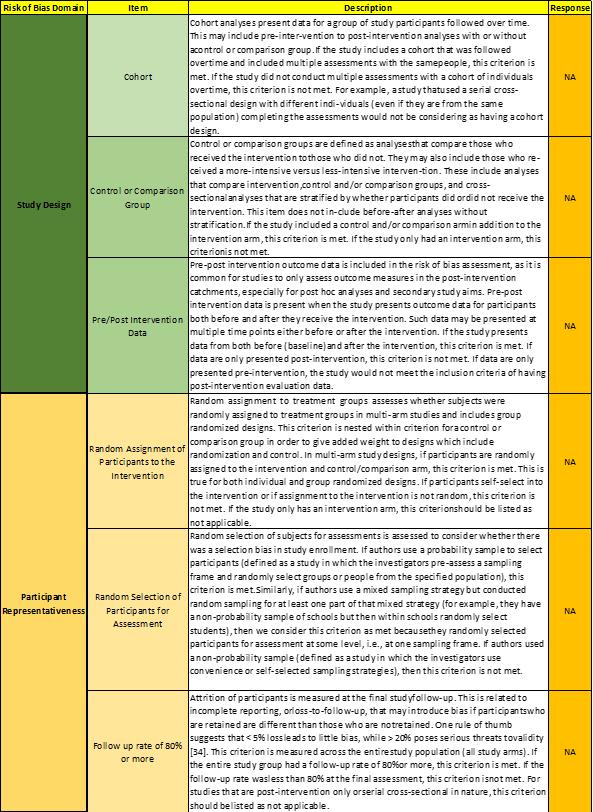


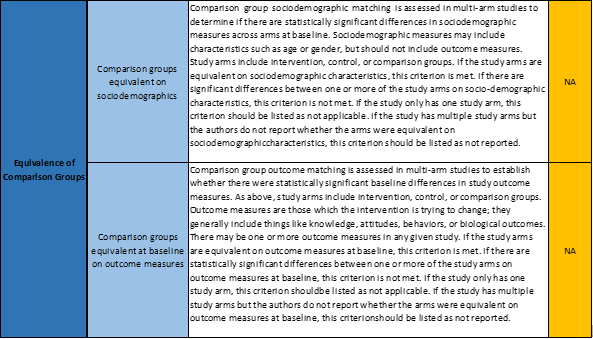


**Figure A2: The Evidence Project Risk of Bias Appraisal Tool Template**

Supplement: Supplementary file 2 — FIGURE A2 The Evidence Project Risk of Bias Appraisal Tool Template [file JSR-32-e13843-s001.docx]
